# Analyzing the Functional Interdependence of Verbal Behavior with Multiaxial Radar Charts

**DOI:** 10.1007/s40614-024-00404-6

**Published:** 2024-04-30

**Authors:** Lee Mason, Maria Otero, Alonzo Andrews

**Affiliations:** 1 Child Study Center, Cook Children’s Health Care System, 1300 West Lancaster Avenue, Fort Worth, TX 76110 USA; 2 Burnett School of Medicine, Texas Christian University, Fort Worth, TX USA; 3 Department of Behavior Analysis, University of North Texas, Denton, TX USA; 4 Professional and Continuing Education, University of Texas at San Antonio, San Antonio, TX USA

**Keywords:** Verbal behavior, Radar charts, Language development, Multiple control, Shape descriptors

## Abstract

The functional analysis of complex verbal behavior requires an evaluation of topographically similar responses under multiple sources of control. Traditional graphical displays of behavior were designed to show the manipulation of isolated controlling variables and may not be amenable to displaying the multidimensional properties of complex behavior. Researchers have recently demonstrated the use of multiaxial radar charts for comparing the functional performance of biological systems. Here we extend the use of multidimensional analyses to compare the relative performance distributions of verbal behavior across four potential controlling variables. First, we provide a conceptual analysis of intraverbal and extraverbal control as continua along which stimuli range from formal to thematic and explain how the intersection of these stimulus fields creates a radar chart for multidimensional analysis. Then we demonstrate how data may be gathered through a verbal operant experimental analysis. We employed repeated measures to map the conditioning history of a child with autism spectrum disorder across 2 years of early intensive behavioral intervention and analyzed the results using shape descriptors for quantitative comparisons. We also compared the polygonal language profiles of children with autism against that of a neurotypical peer. Extending a multidimensional analysis to the field of verbal behavior provides the basis for a language growth chart that researchers and clinicians can use to monitor language acquisition over time. We discuss the use of radar charts as a framework for understanding the interdependence of verbal operants and suggest their use for complex analyses of complex verbal behavior.

**Supplementary Information:**

The online version contains supplementary material available at 10.1007/s40614-024-00404-6.

Skinner’s (1957) *Verbal Behavior* revolutionized the study of human language by providing a novel classification system through which verbal behavior can be analyzed according to distinct functional relations. An extension of his work on operant conditioning, Skinner’s analysis of verbal behavior rebutted contemporaneous approaches to language acquisition by offering an alternative means of observation and measurement. As Skinner grieved, “I have done my share of comma counting” (p. 454).

Applying basic behavioral principles to the field of language development affords a scientific analysis through the systematic manipulation of variables, reproducibility of outcomes, and replicability across studies. Decades of research continue to support the functional distinction of verbal operants, which has been shown effective in remediating language deficits of individuals with autism spectrum disorder (ASD) and other contingency-shaped disorders of verbal behavior (Carr & Miguel, 2013; DeSouza et al., 2017; Sautter & LeBlanc, 2006; Sundberg & Michael, 2001).

In describing the functional distinction of verbal operants, Skinner (1957) contended that the environment can be arranged such that formally similar responses do not readily transfer across operant classes:

The milk which a child gets with the mand *Milk!* resembles the milk which controls the tact *milk* in response to the question *What is that?* This may facilitate the acquisition of whichever operant is acquired second. One could establish the mand *Milk!* through reinforcement with milk as a tactual, gustatory, and olfactory stimulus by feeding the child only from an opaque bottle. At the same time, one could establish a tact of the same form to the visual stimulation of milk in a clear glass. Under these circumstances a child would presumably not show any tendency to transfer the response from one type of operant to the other. (p. 189)

Indeed, such distinct sources of control may prevent the emergence of untrained relations, but the outcomes of studies examining this particular phenomenon have been somewhat inconsistent (Grow & Kodak, 2010; Wooderson et al., 2022). Although some researchers have documented the untrained emergence of mands after tact training (Egan & Barnes-Holmes, 2009; Wallace et al., 2006), other researchers have failed to replicate this effect (Kelley et al., 2007; Pétursdóttir et al., 2005). A complete account of verbal behavior must explain the environmental variables that control emergent responding, as well as the lack thereof.

Different verbal operants are established at different rates (Sundberg & Michael, 2001). Disproportionate levels of strength across the elementary verbal operants may prohibit some speakers from demonstrating the emergence of untrained verbal relations. That is, a relatively strong tact and weak mand repertoire may prohibit the emergence of mand control after tact training, and vice versa.

Proficient speakers display little difficulty in acquiring a novel verbal response under one set of functional relations before emitting it under another. Skinner (1957) described the fluent speaker’s dynamic ability to ebb and flow across unstable environmental variables:

However, a verbal response of given form sometimes seems to pass easily from one type of operant to another. The speaker commonly starts with a tact and then appears to possess a corresponding mand. The child in a toy store, unable to identify a particular toy, asks *What* *is that?* and is told *A doodler*. This is a stimulus for an echoic response—of the sort which is then commonly used to reinforce the response as a tact. But the child immediately says *Buy me a doodler!* He has never been reinforced for this response in the manner required to construct a mand. (p. 188)

The fluency with which a speaker responds to constantly changing environmental conditions is predicated upon a history of abstraction from convergent multiple control (Ferster & Hammer, 1966; Michael et al., 2011). The likelihood of a verbal response may be algebraically strengthened by combining separate controlling relations, such as a toy (i.e., tact control) and the name of the toy (i.e., echoic control). The repeated pairing of both the nonverbal and imitative verbal stimuli, in conjunction with the delivery of reinforcement upon the emission of the toy’s name, increases the strength of both tact and echoic control in isolation. Thus, as environmental variables shift (e.g., the presence and absence of a nonverbal stimuli and imitative verbal stimuli), the speaker’s ability to speak about the toy remains constant. The requisite history of abstracting stimulus control may allow for the emergence of untrained relations when a novel echoic is later emitted as a tact without the explicit conditioning of convergence and abstraction.

A proportionately strong verbal repertoire presupposes the complex history of reinforcement in which verbal behavior is conditioned under abstract stimulus control. Though the controlling relations remain functionally distinct, the emergence of untrained verbal behavior hints at the interdependent nature of verbal operants. Here, we introduce a methodology for evaluating four primary verbal operants as dependent samples by which to measure the complexity of the speaking repertoire. We begin by providing an analogy to other scientific disciplines wherein analyses have evolved from relatively simple to necessarily complex.

## Complex Numbers

Scientists are frequently confronted with the task of simultaneously comparing multiple dimensions of natural phenomena. For instance, questions within the field of mathematics forced a more advanced understanding of complex numbers as the intersection of real and imaginary numbers. At first, counting numbers (1, 2, 3, etc.) were developed as a practical method of observation and measurement. For centuries these numbers were thought to be the extent of mathematics. Only much later was zero created to serve as a placeholder (see Neely, 2012). This set of whole numbers established the groundwork for addition and multiplication.

Whole numbers alone could not provide an adequate basis for subtraction, however, which proved useful to ancient tax collectors who had to find a way to subtract larger numbers from smaller ones. Questions about going into debt led 7^th^-century Indian mathematicians to invent negative numbers. The rules of subtracting and multiplying negative numbers were further developed by Islamic mathematicians, who solved problems with negative coefficients.

Others were not so amenable to this extension of the counting system. It took more than a millennium for the Western world to accept the use of numbers less than zero. Eighteenth-century British mathematician Francis Maseres wrote that negative numbers ". . . darken the very whole doctrines of the equations and make dark of the things which are in their nature excessively obvious and simple" (Rogers, 2009). It was not until John Wallis printed the combination of positive numbers, negative numbers, and zero onto a number line that negative numbers began to be more readily accepted (Heeffer, 2011).

Yet more advanced mathematical questions necessitated an even more complex counting system. Questions about the ratio of one number to another led to the development of rational numbers, which fill the spaces between integers on the number line. The introduction of rational numbers appeared to account for all natural numbers, until the Greeks furthered our understanding of the counting system with their study of square roots. For example, although $\frac{7}{5}$ approximates $\sqrt{2}$, an exact fraction does not exist. Thus, a set of irrational numbers was created to fill the space between fractions on the number line.

Finally, a universal counting system was completed. “It is strange to think of new numbers being ‘discovered,’ but this is mainly because we are so familiar with the numbers we commonly use that we forget that there was a time when some of these numbers were not known,” explained Singh (1997). “Negative numbers, fractions, and irrational numbers all had to be discovered, and the motivation in each case was to answer otherwise unanswerable questions” (p. 81).

**Fig. 1:**
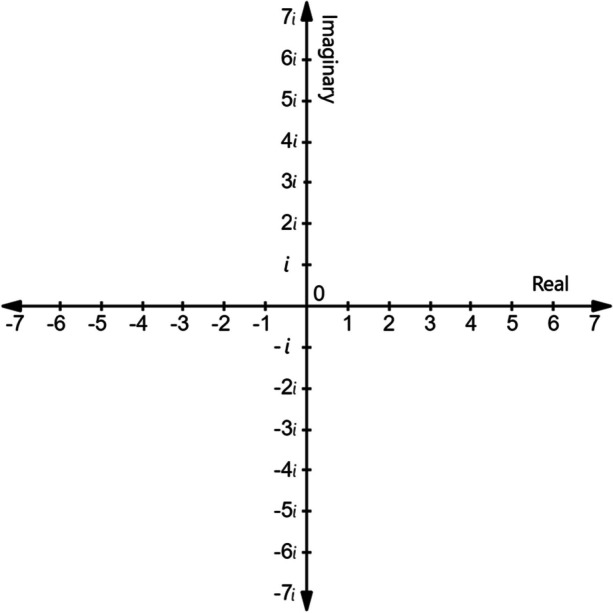
A number plane in which real numbers fall along the horizontal axis and imaginary numbers fall along the vertical axis. Note. The location of complex numbers like 1-3i can be found by counting from zero to the right one and down three

Before long, Italian mathematicians stumbled upon another unanswerable question regarding $\sqrt{1}$, which could be answered as both 1 and -1. But what about $\sqrt{-1}$? Answering this question required the development of imaginary numbers to complement the real numbers already on the number line. For every real number (e.g., -2, $\frac{1}{2}$, 2) an imaginary equivalent exists (e.g., -2i, $\frac{i}{2}$, 2i). Without a natural position for imaginary numbers along the real number line, a separate imaginary number line was created perpendicular to the real one, crossing at zero (see Fig. 1).

This multidirectional coordinate system gave rise to complex numbers consisting of both real and imaginary numbers (e.g., 1-3i). In addition to providing a conceptually complete counting system, the discovery of complex numbers has led to advances in physics, engineering, and economics. Imaginary numbers literally added a new dimension to mathematics, and the invention of the number plane continues to provide a comprehensive foundation for solving complex equations, whereas the intersection of perpendicular axes provides the means for multidimensional analysis (Singh, 1997). Here we extend the use of the number plane to the analysis of verbal behavior. Using each axis of the plane to plot the frequency of topographically similar verbal responses differentiated across sources of control, we create a radar chart for visual and quantitative analysis of the speaker’s verbal repertoire. The dependent sampling of verbal behavior provides a foundation for examining the symbiosis of verbal operants.

## Multiaxial Radar Charts

It is traditional for behavior analysts to use two-dimensional line graphs to display behavior change over time: The x-axis represents the continuum of time, and the y-axis represents the behavior of interest. This type of data visualization is fundamental to analyzing behavior change over time and across different environmental conditions, though limited to depicting a single dimension of behavior. Behavior under multiple sources of control may show greater variability or indistinct data paths on a line graph, which limits interpretation. Moreover, line graphs can be unsuitable for representing the interaction effects of controlling relations.

Lamenting the need to concurrently analyze multiple two-dimensional orthogonal plots, Porter and Niksiar (2018) argued for the use of radial, multi-axis radar charts as a relatively simple and accessible method of displaying the multidimensional performance of mechanical systems on a single graphic across N ≥ 3 properties. A radar chart has three or more axes extending from its origin, such as the number plane described above, and is useful for visually analyzing multivariate data. Porter and Niksiar demonstrated the use of radar charts for: (1) identifying mechanical property-function correlations distinctive to rigid, flexible, and damage-tolerant biological materials; (2) comparing the tensile properties of five collagenous tissues; and (3) demonstrating the trade-off between feeding and singing performance on the beak shape of Darwin's finches. Following the procedures described by Porter and Niksiar for producing geometric profiles, we aim to extend the use of radar charts to analyze functional language development. The radar chart’s ability to depict an array of multidimensional properties may be particularly useful for capturing the interaction of the multiple controlling variables that constitute complex behavior.

Radar charts have been broadly criticized as a data analysis technique, which may explain why behavior analysts have been slow to adopt them. Among the most common reproaches are the overplotting of data, which makes the graphic unreadable (Feldman, 2013); the circular layout, which makes interpretation less accurate (Albo et al., 2015); and the subjective sorting of axes, which can be misleading (Heijungs, 2022).

Despite these criticisms, Porter and Niksiar (2018) explained that the polygonal structure of the radar chart provides a unique platform for quantitative analyses. They conclude that the simple shape descriptors provided by multiaxial radar charts help identify performance trade-offs and profile similarities, and recommend further exploration of their use within the fields of biology and engineering.

As verbal relations lie within a natural science of the behavior of organisms, shape descriptors—like area, centroidal distance, and first moment of area—may be useful metrics for analyzing multiple sources of control. “The basic issue is not the nature of the stuff of which the world is made,” said Skinner (1963), “. . . but rather the dimension of the things studied by psychology and the methods relevant to them” (p. 951). Prior research highlighting the benefits of shape descriptors, along with Skinner’s emphasis on methodology, suggests that radar charts may serve as a convenient platform for a multidimensional analysis of the different environmental relations that control complex verbal behavior.

## Complex Verbal Behavior is Multidimensional

The transformation of the number line into the number plane has direct implications for the study of complex verbal behavior. The science of behavior similarly began with a single line. The cumulative record afforded a quantifiable subset of the organism’s repertoire, making it amenable to quantitative analysis. The two dimensions of frequency and time produced curves that allowed early behavior analysts to interpret environmental relations. Just as unanswerable questions drove the development of a comprehensive counting system, our understanding of emergent language demands similar treatment. Analogous to the complex numbers of a number plane, we present a multidimensional analysis of elementary verbal operants that serves as a foundation for a complete examination of complex verbal behavior under a range of multiple-controlling variables.

Skinner (1953) declared all sustained verbal behavior multiply maintained, encompassing infinite response topographies that occur under evolving circumstances. The compounding effect of interacting variables poses a particular challenge to the scientific investigation of behavior, which led Michael et al. (2011) to suggest multiple control as a useful analytic tool for interpreting complex verbal behavior. The ability to distinguish between functionally independent verbal operants by no means implies that complex behavior can be viewed as an amalgamation of discrete responses. Consequently, it may be more precise to categorize different - and often co-occurring - sources of stimulus control rather than different “types” of verbal behavior.

Though functionally distinct, the operants that comprise an interdependent verbal repertoire cannot be separated from one another. Consider the chemical distinction between a mixture and a compound. A mixture is the physical combination of two or more elements in which no chemical reaction has occurred. Because they have not bonded, the individual substances can be separated into component parts. Saltwater can be separated into salt and water, and air can be separated into various gases. In contrast, compounds are formed by the chemical combination of two or more elements that have bonded together in fixed proportions. The chemical reaction that bonds these pure substances also prevents their physical separation. Hydrogen and oxygen bond to form water, sodium and chloride bond to form table salt, and so too do the elementary verbal operants depend on one another to form complex verbal behavior. Once the convergent control of multiple environmental relations has been reinforced, the integrity of a functionally independent verbal repertoire is no longer preserved, and novel uses may appear to have spontaneously emerged (Gamba et al., 2015).

As described by Skinner (1957), “Separate variables converge to extend their functional control, and new forms of behavior emerge from the recombination of old fragments” (p. 10). It is no longer reasonable to identify a given verbal response as mand *or* tact, but to examine the source(s) of control supporting the response in terms of the degree to which they function under mand or tact control. “Not all stimuli which control operant behavior are single stimuli related to specific performances,” observed Ferster et al. (1975). “In many cases there is a continuous relation between a range of stimuli which control a corresponding range of performances” (pp. 551–552). The response *doll* may involve one or more sources of control emitted in isolation or combination. That is, asking for a doll across the room involves a different set of functional relations than asking for a doll in another room.

### Extraverbal Control

An initial dichotomy can be made between two primary sources of stimulus control over verbal behavior: intraverbal and extraverbal (Vargas, 1982). Extraverbal stimulus control refers to verbal behavior under the control of nonverbal environmental relations. There are two broad categories of extraverbal stimulus control that Skinner (1957) describes as the inverse of one another: “Roughly speaking, the mand permits the listener to infer something about the condition of the speaker regardless of external circumstances, whereas the tact permits him to infer something about the circumstances regardless of the condition of the speaker” (p. 83). The tension between mand and tact controlling relations provides one half of the environmental framework for interdependent verbal behavior.

Mand control benefits the speaker by extending their ability to access reinforcers across time and space. Irrespective of ambient stimuli, mand control denotes verbal behavior under the functional control of the relevant motivating operations. The environmental relations that control a mand are often casually described as *what the speaker wants*. As Hayes (2002) explained, the word *want* comes from the Old Norse term *vant*, which literally translates to *missing*. In other words, the mand occurs in the *absence* of the specified stimulus.

**Fig. 2:**
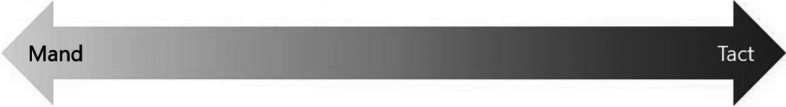
The continuum of extraverbal stimulus control ranges from purely tact in which the presence of a stimulus induces verbal behavior, to purely mand in which the absence of a stimulus induces verbal behavior

Conversely, tact control benefits the listener by extending their contact with the environment across time and space. Tact control denotes verbal behavior uniquely related to a discriminative stimulus without regard for any relevant motivating operations. In this way, the tact denotes the *presence* of the specified stimulus. The symbiotic relationship between speaker and listener points to a continuum of extraverbal stimulus control (see Fig. 2).

We present extraverbal control as a continuum of the range of nonverbal stimuli inducing verbal behavior. The opposing ends of the continuum are represented by mutually exclusive mands and tacts. The preponderance of extraverbal control consists of both, lying somewhere between these two extremes (Bondy et al., 2004; Michael et al., 2011; Skinner, 1957). As in the case of the ancient mariner who pined *Water, water, everywhere,* *Nor* *any drop to drink.* To the extent that a response is more mand, it is correspondingly less tact, and vice versa.

### Intraverbal Control

**Fig. 3:**
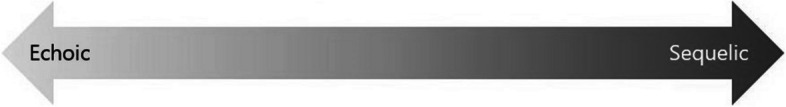
The continuum of intraverbal stimulus control ranges from purely echoic stimuli that induce verbal behavior with exact correspondence, to purely sequelic stimuli that induce verbal behavior without any correspondence

Intraverbal stimulus control refers to verbal behavior under the control of verbal environmental relations (e.g., other people’s verbal behavior). Vargas (1982) further distinguishes between subtypes of intraverbal control: That which evokes verbal behavior with point-to-point correspondence (e.g., echoic), and that which evokes verbal behavior without point-to-point correspondence. To prevent a category error, Vargas introduced the term *sequelic* to describe the latter subtype of intraverbal stimulus control. As with extraverbal behavior, echoic and sequelic are the opposing ends of a continuum of correspondence between verbal behavior and verbal stimuli (see Fig. 3). The tension between echoic and sequelic controlling relations represents the other half of the framework for functional interdependence.

We present intraverbal control as a continuum of the range of verbal stimuli inducing verbal behavior. The ends of the continuum are represented by mutually exclusive echoics and sequelics. The preponderance of intraverbal control consists of both, falling somewhere between these two extremes. As in the case of the Taylor Swift fan who sings about *Starbucks lovers*. To the extent that a response is more echoic, it is correspondingly less sequelic, and vice versa.

These two continua of stimulus control note the extent to which a corresponding extraverbal or intraverbal stimulus is present or absent. Pure examples of the elementary verbal operants may be rare outside of highly controlled laboratory or instructional contexts (Michael et al., 2011). Within ordinary social contexts, the range of controlling variables can be identified by different sources of supplementary stimulation.

### Supplementary Stimulation

Beyond the bifurcation of extraverbal and intraverbal sources, the analysis of controlling properties can be further delineated according to supplemental variables. Skinner (1953) explained that verbal “suggestions” (i.e., prompts and probes) might be classified according to their supplementary sources, and used the terms *formal* and *thematic* to refer to stimuli of the same and different form, respectively. Consistent with the aforementioned analyses of extraverbal and intraverbal control, we once again recognize the false dichotomy of supplementary stimulation. More contemporary discourse surrounding a multiscale view of environmental relations (see Baum, 2018; Hineline, 2011; Rachlin, 2017) allows us to reconceptualize Skinner’s notion of supplementary stimulation in a manner more amenable to multidimensional analysis (Hall & Chase, 1991). Here we use the term formal suggestion to describe the process of explicitly strengthening a response by presenting a verbal or nonverbal supplementary source of control. The observer can point to the specific controlling relations for the given response. Such is the case in advertising through the supplementary use of tact control of product placement (i.e., E.T. eating Reese’s Pieces) or echoic control of a slogan or jingle (e.g., *Better call Saul!*).

On the other hand, thematic suggestion strengthens a response in the absence of any explicit source through derivational supplementary control. The specific controlling relations for the given response are not readily available, though thematically related sources of control are present. For example, why someone would reinforce the sequelic response *Bah Zoo*, when given *Riff Ram* *…,* may be incomprehensible to anybody who did not attend the local university. Likewise, the variables controlling one’s request for *Agua!* after a prolonged exertion may only be apparent after reinforcement of the mand has been mediated.

A cross-classification of verbal behavior may be constructed according to intraverbal or extraverbal stimulus control, and whether the supplementary stimulation is formal or thematic (see Table 1 below). The division of formal and thematic controlling relations identified in the Punnett square represents the two additional endcaps of a continuum of stimulus control. That is, supplementary stimulation may only rarely be purely formal or thematic; more frequently, the source of control lies somewhere in between.

**Table 1:** A Punnett Square demonstrating the cross-categorization of the primary and supplementary variables that control verbal behavior

	Formal	Thematic
Intraverbal	*Echoic*	*Sequelic*
Extraverbal	*Tact*	*Mand*

The resulting categorization of verbal operants yields a subtle, yet important, departure from Skinner’s (1957) taxonomy of verbal behavior, in which formal control refers to the point-to-point correspondence between verbal stimulus and response (cf. Michael et al., 2011). However, we posit that nonverbal stimulus control may also provide a formal source of strength through the minimal repertoire of the tact. Rather than point-to-point correspondence, it is the presence of this minimal repertoire that is the defining property of formal control. The response *dog*, *chien*, or *perro* (among others) are all formally strengthened in the presence of the family pet, and in accordance with the culturally specific reinforcing practices of the verbal community. Whereas *cat*, *fetch*, and *vet* (again, among others) may all be thematically strengthened due to the absence of a minimal unit. The response *bark* will be formally strengthened in the presence of a barking dog and may also be thematically strengthened in the presence of a quiet dog.

A functional analysis of language provides a complete account of the various conditions under which verbal behavior is emitted. Our reframing of extraverbal and intraverbal primary sources across formal and thematic supplementary sources provides the foundation for a multidimensional apparatus examining complex verbal behavior. A single verbal response may be cross categorized by sources of primary and supplementary stimulation. Given the implications of interdependent verbal operants, it is no longer reasonable to consider the verbal repertoire as the mere combination of mutually exclusive component parts. A given verbal response is rarely mand *or* tact, sequelic *or* echoic, but commonly shares the controlling properties of two or more verbal relations. The reinforcement of a verbal response under mand control often resembles the discriminative stimuli of tact control (Skinner, 1957), just as the presence of a reinforcing object is the ideal condition for contacting reinforcement. As our understanding of verbal behavior becomes more complex, our analyses of complex verbal behavior require an appropriate framework for observing and measuring the interaction of multiple, simultaneous environmental influences.

## Analyzing Complex Verbal Behavior

Applying the continua of intra- and extraverbal sources of control described above, we can construct a plane for analyzing complex verbal behavior similar to that used for analyzing complex numbers. Extraverbal (i.e., tact and mand) sources of control appear along one stimulus continuum, whereas intraverbal (i.e., echoic and sequelic) sources of control appear along the other.

**Fig. 4:**
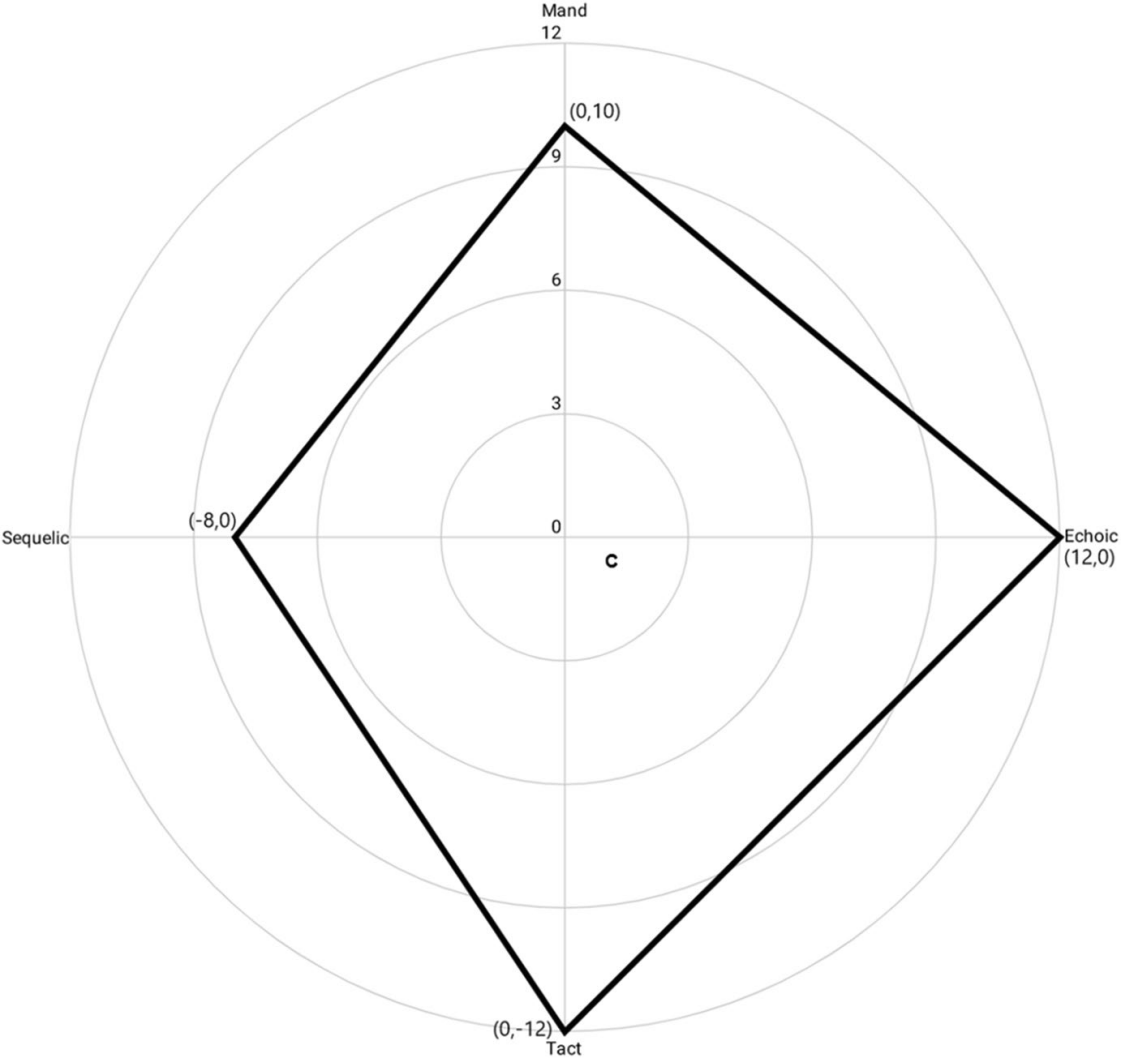
Connecting the plotted data on a radar chart creates a closed polygonal language profile upon which shape descriptors can be calculated. Note. “c” denotes the centroid of the polygonal language profile, located at (1.33, -0.67)

The intersection of these perpendicular lines forges the crosshairs of a radar chart for analyzing four different dimensions on a single graphic. Connecting the plotted data for responses under tact, echoic, mand, and sequelic stimulus control forms a closed polygonal language profile (PLP) of precise size, position, and shape open to geometric interpretation (see Fig. 4).

The PLP in Fig. 4 displays a multidimensional representation of the speaker’s verbal repertoire. The frequency of responses for each operant is recorded on each of the respective axes, with smaller values closer to the origin and larger values further away. A straight line is then drawn between adjacent data points to construct an individualized PLP. Polygonal language profiles are unique characterizations of a speaker’s verbal repertoire that provide an innovative means of visual and quantitative analysis.

The space between adjacent axes represents convergent multiple control of these two variables. Using Fig. 4 as an example, quadrant 1 shows the convergence of echoic and mand control, quadrant 2 shows the convergence of mand and sequelic control, quadrant 3 shows the convergence of sequelic and tact control, and quadrant 4 shows the convergence of tact and echoic control. Quadrants containing greater amounts of the PLP are indicative of greater convergent multiple control. Conversely, quadrants containing less of the PLP indicate a smaller degree of convergent control. Visual analysis of Fig. 4 indicates the convergence of tact and echoic control (quadrant 4) as the greatest source of strength, followed by echoic and mand control (quadrant 1), then sequelic and tact control (quadrant 3), and finally mand and sequelic control (quadrant 2).

### Shape Descriptors

**Fig. 5:**
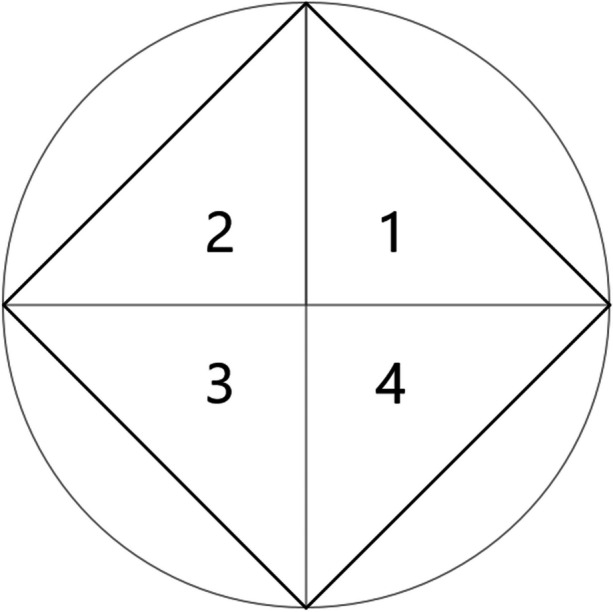
The segmentation of a quadrilateral into four right triangles. Note. The four right triangles are numbered according to the four quadrants of the coordinate plane. The circle surrounding the rhombus represents the property space along which the multidimensional performance of a system is measured

Each radar chart produced a novel PLP that was used to calculate shape descriptors. Just as statistical moments are used to describe a probability distribution, shape descriptors are a set of computational tools used to describe a physical quantity. Shape descriptors—such as size, coordinates, and orientation—are all moment-based attributes (Leu, 1991). Each of these mathematical functions produces a quantitative value that serves as a parameter of the given shape. Once an initial shape descriptor has been calculated, it may be used for additional, more complex computations. Following the procedures of Porter and Niksiar (2018), we calculated four shape descriptors for each PLP produced by the radar chart: area, the total size of the PLP; centroid, the geometric center of the PLP; centroidal distance, the distance from the origin of the chart to the centroid of the PLP; and first moment of area, the spatial distribution of the PLP in relation to the origin of the chart. Although Porter and Niksiar employed a custom MATLAB routine for identifying shape descriptors, we opted for a more straightforward approach that computes the same set of shape descriptors using only the corner pixels along the shape's boundary (Leu, 1991). The basic strategy was to construct a set of four right triangles using the shape's corners and the origin of the coordinate system (see Fig. 5). Computing these four triangles’ descriptors allowed us to derive the moments of the larger shape.

For each PLP, we began by calculating the area (*A*) of the geometric profile it produced using formula 2:

1 $$A=\frac{1}{2}\left(\frac{\left|{x}_{1}\right|}{\left|{y}_{1}\right|}\frac{\left|{x}_{2}\right|}{\left|{y}_{2}\right|}+\frac{\left|{x}_{2}\right|}{\left|{y}_{2}\right|}\frac{\left|{x}_{3}\right|}{\left|{y}_{3}\right|}+\frac{\left|{x}_{3}\right|}{\left|{y}_{3}\right|}\frac{\left|{x}_{4}\right|}{\left|{y}_{4}\right|}+\frac{\left|{x}_{4}\right|}{\left|{y}_{4}\right|}\frac{\left|{x}_{1}\right|}{\left|{y}_{1}\right|}\right)$$

where the vertices are expressed in terms of the absolute value of Cartesian coordinates (*x , y*). Using Fig. 4 as an example, we calculate *A* by inserting the four points into Eq. 2. Each pair of coordinates represents the four quadrants of the coordinate plane. Beginning with quadrant 1, we worked counterclockwise around the four quadrants.

2 $$A=\frac{1}{2}\left(\frac{\left|12\right|}{\left|0\right|}\frac{\left|0\right|}{\left|10\right|}+\frac{\left|0\right|}{\left|10\right|}\frac{\left|-8\right|}{\left|0\right|}+\frac{\left|-8\right|}{\left|0\right|}\frac{\left|0\right|}{\left|-12\right|}+\frac{\left|0\right|}{\left|-12\right|}\frac{\left|12\right|}{\left|0\right|}\right)$$

For each quadrant, we use the absolute value of the length of x axis as the base and the absolute value of the length of the y axis as the height.

3 $$A=\frac{1}{2}\left(120+80+96+144\right)=220$$

This process is repeated for each configuration of axes, targeting the maximal total area ($\widetilde{A}$):

4 $$\widetilde{A} =max{\sum }^{n}A$$

The profile area was then used to calculate three other geometric descriptors: centroid, centroidal distance, and first moment of area. The centroid, or geometric center of a mass, is the arithmetic mean position of all the points in the figure. Similar to the procedure for calculating area, we calculated centroid by dividing the polygon into four right triangles using the shape's corners and the origin of the coordinate system. From the component parts for each individual triangle—area (*A*_*i*_), centroidal distance from the x-axis (*x*_*i*_), and centroidal distance from the y-axis (*y*_*i*_)—we then used the following formulas to calculate the entire polygon’s Euclidean distance from the horizontal axis:

5 $$\overline{x } = \frac{\sum {A}_{i}{x}_{i}}{\sum {A}_{i}}$$

and vertical axis:

6 $$\overline{y } = \frac{\sum {A}_{i}{y}_{i}}{\sum {A}_{i}}$$

respectively. This allowed us to pinpoint the polygon’s centroid using the Cartesian coordinates of $\overline{x }$ , $\overline{y }$.

Having found the centroid, we then calculated the centroidal distance (*R*) from the origin of the coordinate system:

7 $$R=\sqrt{{\overline{x} }^{2}+{\overline{y} }^{2}}$$

**Fig. 6:**
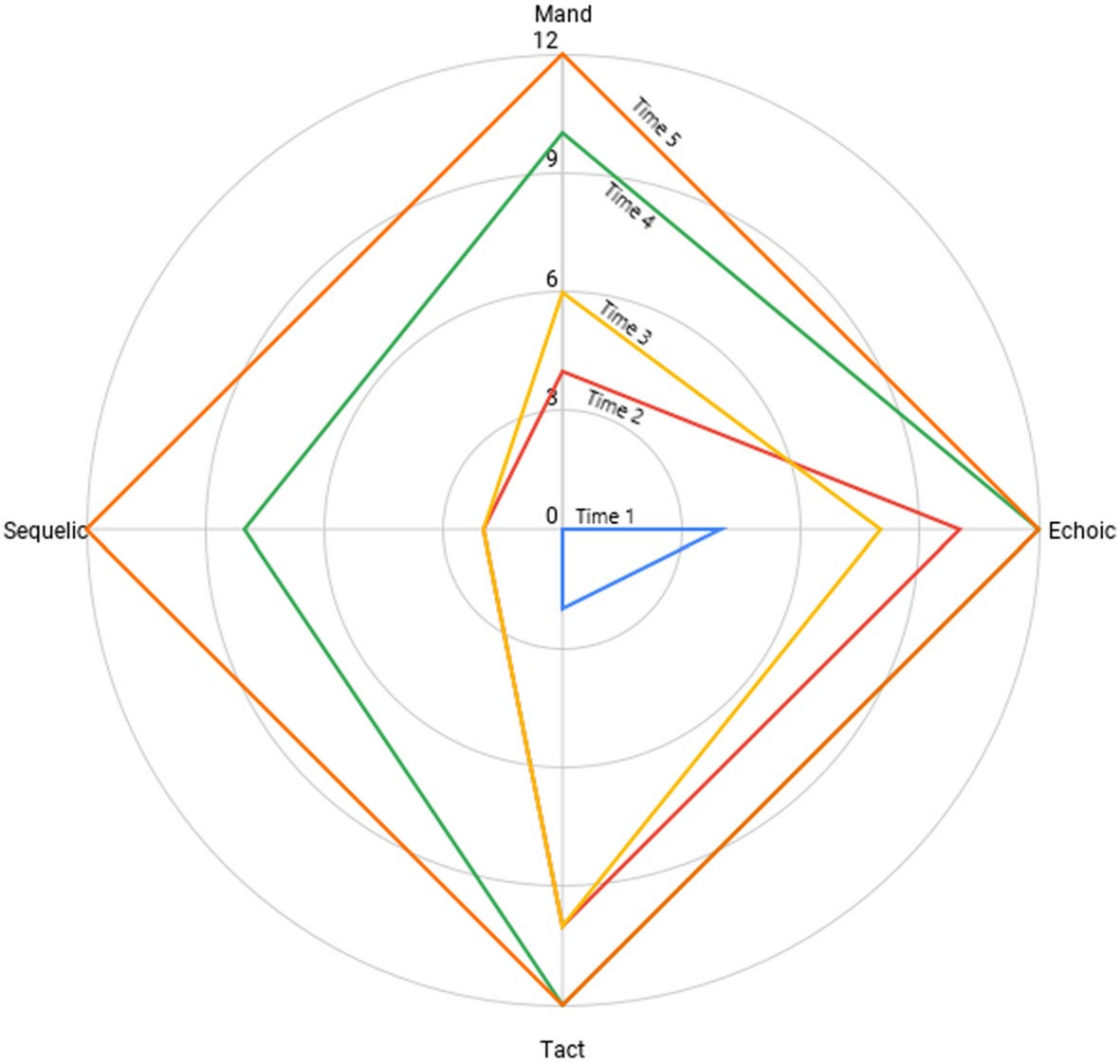
A radar chart showing the language development of a child with ASD from 3 to 5 years of age. Note. A novel VOX analysis was conducted twice yearly. The overlapping displays prohibit the plotting of centroids

Finally, with all the prerequisite variables, we were able to calculate the first moment of area (*Q*) for the PLP on the radar chart by subtracting the centroidal distance (*R*) from the radius of a circle (*C*) that circumscribes the property space of the polygon (see Fig. 6), and multiplying the difference by the area (*A*) of the polygon:

8 $$Q = A(C-R)$$

This metric is much like the quantitative measure of inertia within the field of physics or the characteristics of a distribution function within the field of statistics (Flusser et al., 2009). By analogy, Porter and Niksiar (2018) define the relative multidimensional performance of a system as its profile’s “first moment of area relative to the boundary of the property space” (p. 5).

## Verbal Operant Experimental Analysis

Skinner (1957) questioned whether a verbal response’s ability to pass easily from one type of operant to another represented the spontaneous origin of an operant. “The behavior of ‘asking for the word needed to ask for a toy’ is a mand reinforced by (and hence specifying) auditory behavior on the part of the listener which, when echoed characteristically produces the toy,” he explained. “Once this has happened, the response exists as an independent mand because it has been reinforced as such” (p. 188). Skinner’s taxonomy requires knowledge of the circumstances under which a response is emitted. For example, “*Fire* may be (1) a mand to a firing squad, (2) a tact to conflagration, (3) an intraverbal response to the stimulus *Ready, aim . . .* or (4) an echoic or (5) textual response to an appropriate verbal stimuli” (p. 186). The novel usage of a verbal response does not arise spontaneously. It can occur only when the individual operants comprising the verbal repertoire are strong enough to support such relational flexibility (O’Toole et al., 2009). The functional interdependence of the verbal operants supports Michael et al.’s (2011) assertion that multiple control over verbal behavior is the rule rather than the exception.

To demonstrate the utility of multidimensional performance mapping, we extended the above-described shape descriptor analysis to the archival verbal behavior records of a male child diagnosed with ASD. From the ages of 3 to 5, this child’s functional language skills were evaluated every 6 months using a verbal operant experimental (VOX) analysis to sample his verbal repertoire (Mason & Andrews, 2019). An extension of Lerman et al.’s (2005) functional analysis of verbal behavior, a VOX analysis is formalized for statistical analysis (Davison, 1999).

The properties of a stimulus which are relevant in evoking a response, either in the individual speaker or according to the practices of a given community,” proclaimed Skinner (1957), “can be discovered only by considering a series of occasions upon which the properties are systematically varied and the presence or absence of the response noted” (p. 117). Prior research employing functional analyses of verbal behavior focused on a particular response topography determined a priori. Normand et al. (2008) suggested, “Future research should evaluate ways to produce similar rates of verbal responding across experimental conditions while differentiating the response form observed, especially if empirical support for the general validity of Skinner’s analysis is to be inferred” (p. 66). In a VOX analysis, a speaker-selected set of verbal responses are each assessed under pure^1^ sources of mand, echoic, tact, and sequelic control. By controlling for potential confounding variables, researchers can begin to compare the proportionality of the speaker's repertoire relative to different sources of control across topographically similar verbal responses.

The analysis begins with a free-operant preference assessment in which the speaker chooses from a range of preferred items. The free-operant preference assessment serves three important functions. First, a sampling of idiosyncratic responses is critical to eliminate systematic bias (e.g., selecting items related to the verbal responses frequently emitted by the speaker), and is an essential assumption of quantitative modeling. The preference assessment also references the speaker’s history of conditioning, providing a basis for later probing sequelic responses. For example, rolling a ball down a ramp may occasion the frame *You roll the. . . .* Finally, allowing the speaker to label each item is responsive to speakers from culturally and linguistically diverse backgrounds. Given that a single stimulus controls a variety of responses (Michael et al., 2011), the tact condition allows the speaker to produce a response consistent with the reinforcing practices of their own verbal community.

Once an item has been selected, tact control is assessed by pointing to the item in the speaker’s possession and asking them to label it. Although assessing tact control, careful consideration must be taken to control for mand, echoic, and sequelic confounds. This process is then repeated until the speaker’s ability to label has been assessed for three unique verbal responses. A unique feature of assessing tact control in this way is that it allows the speaker to select the form of the response, and is therefore culturally responsive to heritage languages, neologisms, and approximations emitted by the speaker. The responses emitted by the speaker throughout the tact condition are then used as targets when assessing mand, echoic, and sequelic control.

The three items identified in the tact condition can then be assessed for mand function using a multiple stimulus without replacement (MSWO) preference assessment. The three items are placed in front of the speaker, who is asked to select one. Although the speaker is engaging with the selected item, the other two are removed from the stimulus field. Access to the target object is then restricted, inducing the speaker to request it back. Although assessing mand control, careful consideration must be taken to control for tact, echoic, and sequelic confounds. This process is then repeated until the speaker’s ability to request has been assessed for all three target responses.

Echoic and sequelic control can both be assessed using discrete trials. In the echoic condition, each of the responses provided by the speaker during the tact condition is presented as an imitative verbal stimulus. Although assessing echoic control, careful consideration must be taken to control for tact, mand, and sequelic confounds. This process is then repeated until the speaker’s ability to echo has been assessed for all three target responses.

In the sequelic condition, a unique fill-in-the-blank frame or Wh- question is created to direct the speaker to respond with the name of each stimulus (e.g., *Quack, quack says the . . . *, or *What* *says quack quack?*). Although assessing sequelic control, careful consideration must be taken to control for tact, mand, and echoic confounds. This process is then repeated until the speaker’s ability to converse has been assessed for all three target responses.

It is notable that “The ‘word’ as a unit of analysis is appropriate to the practices of the *community* rather than the behavior of the individual speaker” (Skinner, 1957, p. 190). The same three verbal responses are equally assessed across the four conditions of the VOX analysis, and the frequency of responses recorded for each operant class. The entire process may be repeated until a sufficient sampling of the speaker’s verbal repertoire has been obtained for a comparison of percentages in related samples to test the functional independence of verbal operants (i.e., significant at the .05 level; Mason et al., 2022). Though each round of the VOX analysis begins with an assessment of tact control, the remaining order of the conditions is randomized to control for sequencing effects.

Table 2 displays the tabular data obtained from a VOX analysis of a 3-year-old boy with autism. All six units of response were assessed across each source of control in semi-random order, and the presence or absence of the response was recorded by the listener. The total frequency for each operant was then plotted on the corresponding axis of a radar chart, with each of the four axes representing one of the four assessed verbal operants. Area, centroidal distance, and first moment of area were calculated using the methods described above.^2^

**Table 2:** The raw data from a VOX analysis of a 3-year-old boy diagnosed with autism spectrum disorder

Response	Tact	Mand	Echoic	Sequelic
“iPad”	0	0	1	0
“Guitar”	1	0	1	0
“Spider”	1	0	1	1
“Lion”	1	1	1	0
“Phone”	1	0	0	0
“Step on it”	1	1	1	0
Total	5	2	5	1

Each verbal response was probed as four distinct verbal operants. For example, the response, “Step on it,” was emitted while the speaker held a rubber floor dot (tact), and subsequently assessed under mand, echoic, and sequelic control. A “1” indicates the response was emitted for the respective source of control, while a “0” indicates the response was not emitted. At the end of the assessment, the summed frequency for each column was plotted on a radar chart for visual and quantitative analysis

**Table 3:** The different profile properties of a child with ASD, Whose language was assessed Biannually between the ages of 3 and 5 years

	Time 1	Time 2	Time 3	Time 4	Time 5
Age (YY:MM)	3:4	4:0	4:5	4:11	5:5
Area (*A*)	4	84	80	220	288
Centroidal Distance (*R*)	1.49	3.33	2.40	1.49	0.00
First Moment of Area (*Q*)	42.04	728.00	767.70	2312.04	3456.00

### Functional Distribution

Porter and Niksiar (2018) explained that the multidimensional analysis of a radar chart allowed for the functional grouping of similar properties, as in a finch’s ability to eat and sing. Here we find that the maximum-area plot centralizes the point of convergent control over the speaking repertoire. One advantage of calculating the first moment of area is that it allows us to identify the centroid of a given shape. The centroid is the barycenter of the four verbal operants and provides a measure of the verbal repertoire’s locus of control.

Skinner (1974) described the locus of control as the intersection of an organism’s genetic endowment, history of reinforcement, and current environmental antecedents. Each of these three elements is accounted for in a functional analysis of verbal behavior. One’s genetic endowment refers to the individual’s mode of communication (e.g., vocal, manual sign, speech-generating device). Their history of reinforcement accounts for operant strength and other variables like language conventions. Meanwhile, the environmental antecedents are systematically controlled throughout the functional assessment. Far from a mere construct, radar charts allow us to pinpoint the locus of control over a speaker’s verbal repertoire. A perfectly balanced speaking repertoire (i.e., an equivalent response rate observed across all operants) has a centroid of 0, 0. Centroidal distance refers to a hypothetical line drawn from the origin of the radar chart to the centroid and is factored into the first moment of area. We posit that a verbal repertoire with a centroidal distance closer to the origin of the radar chart is more sensitive to changes in the environment. A small centroidal distance is a critical prerequisite to the emergence of untrained relations. Conversely, a large centroidal distance is consistent with descriptions of restricted stimulus control over the verbal behavior of speakers with ASD (Mason et al., 2022; Atherkode & Mason, 2023).

### Observing Change over Time

Like traditional line graphs, radar charts provide the ability to observe change over time, while minimizing the probability of overplotting data. Figure 6 shows the results of five VOX analyses conducted with the same speaker over 2 years at 6-month increments. Visual analysis shows the expansion of the verbal repertoire across all four operants over time, as well as the shifting of the centroid toward the intersection of the axes.

Depending on the alignment of their boundaries, it may be difficult to compare more than four or five series of data on a radar chart. Figure 6 displays five series because they are largely dispersed over the area of the chart. When there are large amounts of overlap, it may be more appropriate to limit the number of series.

For example, the large degree of overlap between the boundaries of Times 2 and 3 may justify removing the other series to allow for a more detailed comparison. It is notable that the calculation of the area of the speaker’s verbal repertoire at Time 2 was larger than that at Time 3, but the smaller centroidal distance at Time 3 yielded a greater first moment of area (see Table 3).

**Fig. 7:**
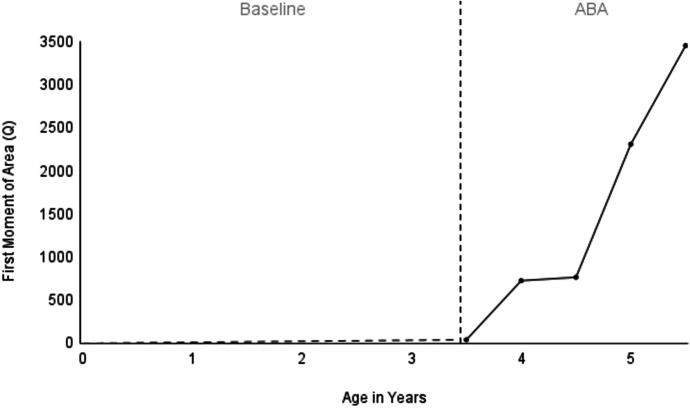
A time-series graph showing the language development of a young boy diagnosed with ASD. Note. The baseline data are dashed to note a presumed language trajectory, as his parents reported no regression of language skills

This child’s language acquisition is clearly displayed across each 6-month interval on the radar chart, and precisely quantified by the data in Table 3. The longitudinal results for first moment of area (*Q*) can also be plotted on a line graph to examine level, trend, and variability (see Fig. 7). Given that *Q* is itself a quantification of the interaction between intraverbal and extraverbal control, the addition of time on the abscissa produces a three-dimensional model expressed within a two-dimensional space.

Another advantage of the radar chart is that it allows for the comparison of language profiles across speakers. Figure 7 shows the language profiles of four different children. Charts A and B show greater similarities in the size and shape of their language profiles, when compared against those of charts C and D.

**Fig. 8:**
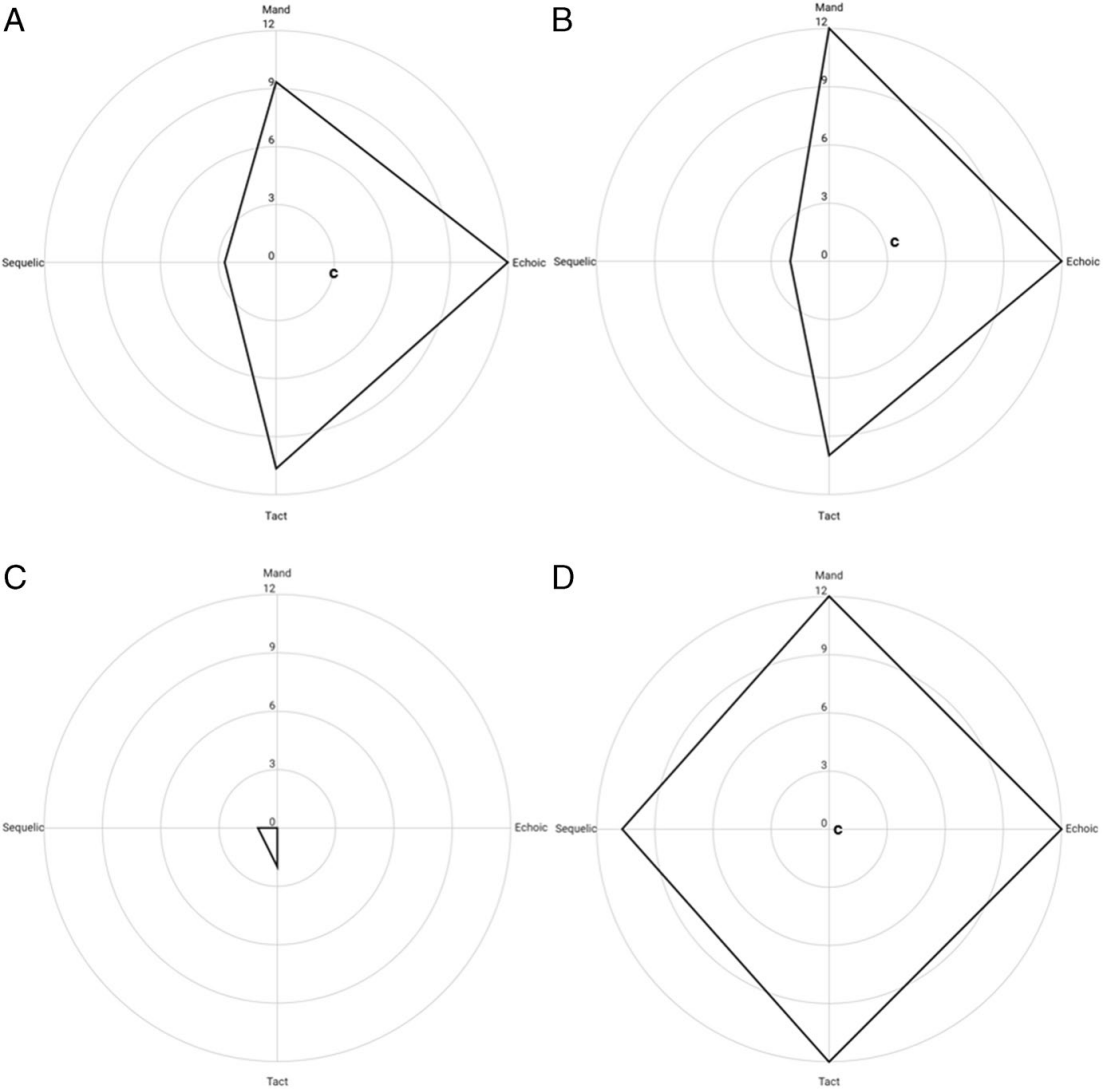
A comparison of polygonal language profiles from four different speakers. Note: Child A (upper left) was a 4-year-old boy with ASD, centroid (“c”) is (3.0, -0.5); Child B (upper right) was a 6-year-old girl with ASD, centroid is (3.33, 0.67); Child C (lower left) was a 4-year-old girl with ASD, centroid (not shown) is (-.33, -.67); and Child D (lower right) was a neurotypical 3-year-old boy, centroid is (0.5, 0.0)

The multidimensional axes allow for a more straightforward interpretation of similarities and differences across individuals. Three of the four charts in Fig. 8 (A, B, and C) were gathered from the language samples of children diagnosed with ASD, whereas Child D showed typical language development. The characteristics of these children are displayed in Table 4. The similarities of geometric language profiles of children A and B may lead to similar courses of treatment or instructional grouping, such as a general focus on conditioning intraverbal control. Likewise, the nuanced difference between these profiles (e.g., location of the centroid) may point to subtly different courses of action, such as the specific prompt hierarchy used with each child.

**Table 4:** A comparison of the geometric language profile properties of four children

	Chart A	Chart B	Chart C	Chart D
Sex	Male	Female	Female	Male
Race/Ethnicity	African American/ Non-Hispanic	White/Hispanic	African American/ Hispanic	White/Hispanic
Home Language(s)	English	English/Spanish	English	Spanish
Age (YY:MM)	4:5	6:0	3:9	3:10
Area (*A*)	146.67	154.00	1.00	272
Centroidal Distance (*R*)	3.14	3.40	0.75	0.44
First Moment of Area (*Q*)	1299.07	1324.50	11.25	3143.11

Child C’s language profile is drastically different from the first two. Although tact and sequelic control exist at some strength, she displayed no functional ability to request or echo. In contrast with Children A and B, Child C would likely benefit most from the explicit conditioning of distinct verbal operants.

Finally, Child D demonstrates the language profile of a neurotypical 3-year-old boy. Unlike the other profiles, Child D’s language shows proportionate levels of strength, with no significant differences among the four verbal operants (Mason et al., 2022). The dynamic ability to alter one’s verbal behavior in accordance with changing environmental conditions—such as Skinner’s (1957) depiction of a child in a toy store—is a function of a relatively large and balanced PLP. Though only 1 month older than Child C at their respective time of assessment, the language profiles of these two children represent opposite ends of the spectrum.

### The Context for Verbal Behavior

In describing his concept of umwelt, von Uexküll (1934/2013) delineated an organisms’ *surroundings*, the items within its stimulus field, from its *environment*, those to which it responds. Perhaps a similar distinction is necessary to differentiate the environmental control over verbal behavior from the context in which it is emitted. Although it is granted that proximate and distal environmental relations account for verbal behavior, here we use the word *context* to describe the strength of a given operant relative to the rest of those that comprise the speaker’s verbal repertoire.

**Fig. 9:**
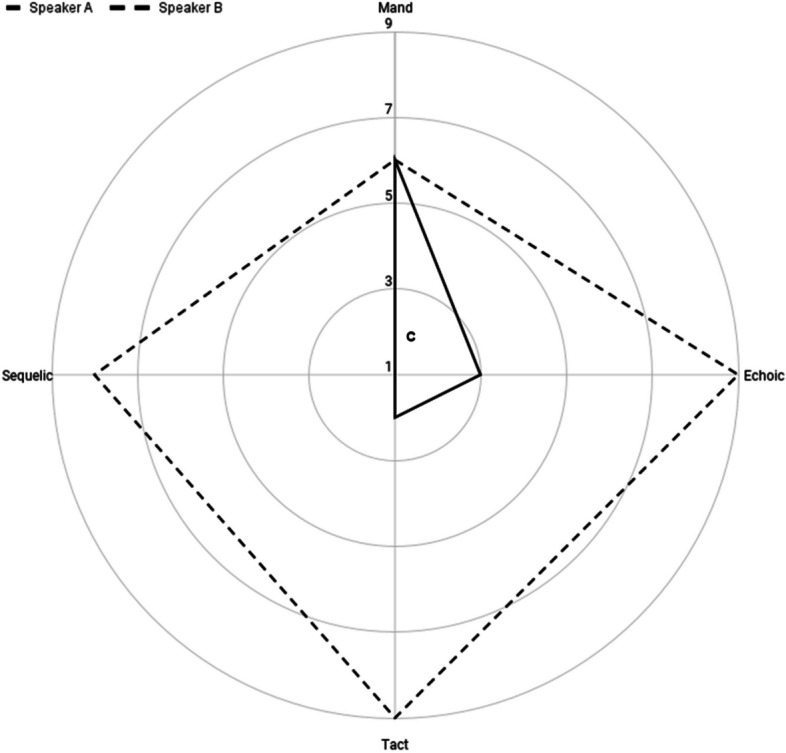
A comparison of two speakers who demonstrate the same level of mand control over vastly different verbal repertoires. Note. “c” denotes the centroid of Speaker A’s polygonal language profile, located at (1.33, 1.78). The centroid for Speaker B’s polygonal language profile (not shown) is (0.44, -1.33)

Figure 9 depicts two verbal repertoires on a single radar chart. Speaker A’s VOX analysis shows a mand value of six within the context of a relatively small verbal repertoire, whereas Speaker B’s VOX analysis shows a mand value of six within the context of a relatively large verbal repertoire. In an absolute sense, both speakers display sequelic responding of identical strength. In the applied setting, however, these profiles represent two uniquely individual speakers that likely have different treatment objectives, interventions, and prognoses.

Skinner’s (1957) notion of functionally distinct verbal behavior led to a largely molecular study of verbal operants, which has undoubtedly had an impact on individuals with functional language disorders. As questions about language development become more complex, so will their answers. We propose the use of radar charts for analyzing component parts within the context of the multidimensional whole. Perhaps this will lead to an understanding of why some individuals continue to require explicit conditioning across verbal operants, whereas others demonstrate the emergence of untrained verbal behavior.

### Compatibility with Other Sciences

The advantages of multiaxial radar charts have immediate application for behavior analysts examining functional interdependence. Conceptually systematic with Skinner’s (1957) analysis, we propose the dichotomous variables of nonverbal (mand-tact) and verbal (echoic-sequelic) stimulus control as a framework for examining complex language development. Extending Porter and Niksiar’s (2018) multidimensional analysis of biological systems to the field of verbal behavior introduces a methodology for visually analyzing simultaneous sources of control. In addition, the closed PLP that results from the multiaxial radar chart allows shape descriptors for precise quantitative measurements and comparisons.

Using radar charts to analyze complex language brings behavior analysis into closer contact with other natural sciences, such as geometry and physics. As noted above, the PLP of the radar charts lend themselves to simple shape moments and other geometric descriptors. Shape descriptors are frequently used in image analysis for identifying similarities between the features of different shapes. Peura and IIvarinen (1997) argued that simple shape descriptors have the advantages of faster calculation and general applicability. Here we introduce area and centroid as two relevant descriptors of complex verbal behavior because of their intuitive extrapolation. Larger areas correspond with greater stimulus control, and smaller centroidal distances equate to higher multifunctionality. Other descriptors may have different interpretations, but Porter and Niksiar (2018) “caution against the use of arbitrary descriptors with little to no explicable abstraction” (p. 12). Future research on shape descriptors of verbal behavior should seek to examine the utility of other primitive shape descriptors, like convexity, compactness, and variance. Given their use in image processing, it is easy to see the implications of verbal behavior shape descriptors on machine learning and data mining of large data sets.

Earlier we delineated between physical and verbal stimuli to differentiate between extraverbal and intraverbal control, but we would be remiss not to clarify that verbal stimuli are physical too. To date, the physical science of language has largely been limited to properties like sound waves (Magrassi et al., 2015) and syntax (Ramgoolam et al., 2022). The geometric language profile of a radar chart affords a novel dimension for physical investigation. Here we extended the use of first moment of area to measure complex verbal behavior. In physics and mechanics, moment is the rotational complement of linear force, also known as torque. Two children can balance on a seesaw because they are of equivalent weight and distance from the fulcrum. An adult replacing one of the children would need to sit closer to the fulcrum to achieve moment balance.

Varignon’s *Principle* *of Moments* states that when multiple coplanar forces act in equilibrium, the algebraic sum of their moments about a given point (e.g., the centroid) is equal to the moment of their resultant force about the same point. One can easily see the parallels between this law of physics and Skinner’s (1938) *Law of Algebraic Summation*, which states that “a stimulus which simultaneously evokes two or more responses in opposite directions produces behavior the extent of which is an algebraic resultant” (p. 30). Skinner (1957) explicated the application of this principle to verbal behavior:

Neither the fact that a single response may be controlled by more than one variable nor the fact that one variable may control more than one response violates any principle of scientific method. . . . These two facts make it highly probable that any sample of verbal behavior will be a function of many variables operating at the same time. Any response under the control of one variable has a fair chance of being related to other variables also present. Now, it is a well-established principle in nonverbal behavior that separate sources of strength are additive. (Because some variables *reduce* the strength of verbal behavior, the addition must be algebraic; p. 228.)

Though the subject matter differs, Varignon’s Theorem is directly analogous to Skinner’s. The multiaxial radar chart clarifies the relationship of both principles to complex verbal behavior and alludes to future investigations of higher-order moments (e.g., moment of inertia).

## Conclusion

Although the focus of this paper has been on demonstrating the utility of multiaxial radar charts for understanding complex verbal behavior, researchers should consider extending these procedures to other explorations of multiple control. Most notable, functional analyses of challenging behavior often point to multiple maintaining contingencies. Radar charts may also be helpful for examining preference assessments, conditional discriminations, and composite behaviors like reading and other academic skills.

Although basic researchers have demonstrated and documented Skinner’s (1938) law of summation, applied research in the field of verbal behavior is nascent (Oliveira et al., 2023). The algebraic summation of controlling variables is fundamental to analysis presented herein, as the purity of each verbal operant is only assessed at the extreme. Future research must seek to validate the assumptions of summation.

Our extrapolation of functional interdependence as abstraction from convergent multiple control also demands empirical justification. Additional research in this area is necessary to show multiple control as a prerequisite to emergent verbal behavior. Future research should aim to demonstrate the functional relationship between abstraction and functional interdependence.

Furthermore, our radar-chart analyses lead us to contend that individuals with ASD may have difficulty responding to compound antecedent stimuli across operant classes. As noted above, centroidal distance can be a useful measure of stimulus overselectivity. Behavior analysts are uniquely positioned to validate this assumption through empirical measures that confirm or contradict stimulus prepotencies identified within a speaker’s VOX analysis.

Practitioners need not despair at the prospect of calculating moments of area and centroids, as this is efficiently accomplished through the use of spreadsheets or statistical software. For those considering adopting this assessment technology, we have created a ShinyApp^3^ (v1.8.1.1; Chang et al., 2024) based on R Statistical Software (v4.3.3; R Core Team, 2024); a link to which can be found in the attached supplemental material. We encourage interested readers to use the software with their own data and send us your feedback.

The multidimensional analysis presented here is consistent with the multidimensional scaling proposed as a model for analyzing derived relational responding and evaluating relational coherence (Belisle & Clayton, 2021; Clayton & Hayes, 2004; Mason et al., 2024). Just as we have extended the principles of shape descriptors and graph theory to the study of verbal behavior, relational density theory posits that relational networks follow the basic physical properties of density, volume, and mass. Moreover, just as we contend that a multidimensional analysis of verbal behavior may lead to higher-order moments, relational density theory proposes higher-order properties of relational acceleration and gravity (Belisle & Dixon, 2020). There is clear alignment between these two frameworks to urge future researchers to approach the study of complex verbal behavior using the laws of physics.

Here we have proposed the use of multiaxial radar charts as a framework for analyzing complex verbal behavior. Radar charts not only provide a unique form of visual analysis, they also allow researchers and practitioners to quantify the precise size of the verbal repertoire as first moment of area (*Q*). As an analytic tool, the radar chart may provide a framework for further studying certain aspects of language development, such as multiple control and functional interdependence.

As noted by Michael et al. (2011), “The simplicity of a principle does not protect us from the complexity of nature” (p. 3). An analysis of functional interdependence is necessary for a complete understanding of complex verbal behavior. Although multiple control does not presuppose the functional independence of the elementary verbal operants, it may obfuscate their uniquely controlling relations. The only way to achieve composite verbal behavior is by first conditioning the relevant component skills. Indeed, the whole is greater than the sum of its parts. A complete understanding of how functionally distinct atomic repertoires comprise complex verbal behavior requires a method for recording and measuring multiple-controlling variables (Palmer, 2012). Though multiple convergent control is never directly assessed, the notion of functional interdependence is premised upon an adequate sampling of the same verbal responses occurring across operant classes. Future research should examine the extent to which a VOX analysis accurately employs Skinner’s (1938) law of algebraic summation to predict the strength of convergent multiple control.

Although Skinner’s analysis omitted several steps between an infant’s babbling and a Shakespearean sonnet, Sturdy and Nicoladis (2017) implore language-development researchers to exhaust the framework of operant conditioning before proposing novel constructs. As other researchers have noted, achieving practical control over the individual operants of a developing verbal repertoire is a prerequisite to achieving practical control over complex verbal behavior (Pétursdóttir, 2018). Because the complex repertoire develops through successive approximations, it is not easily undone. The complex history of reinforcement cannot be forgotten.

The elementary verbal operants provide sufficient explanatory power for the development of complex verbal behavior, but a more sophisticated analytic method may be necessary for its prediction and control. The conventional, two-dimensional approach to measuring the strength of a given verbal operant may be insufficient for observing and recording complex language. Instead, we have proposed using multiaxial radar charts for this purpose. A precise measure of functional interdependence is essential to a scientific investigation of complex verbal behavior.

## Supplementary Information

Below is the link to the electronic supplementary material.

Supplementary file1 (DOCX 27 KB)

## Data Availability

All data generated or analysed as part of this study are included within the published article.

## Footnotes

1 As Skinner (1966) explains, “The ultimate test of uniformity or reproducibility is not to be found in the methods used but in the degree of control achieved, a test which the experimental analysis of behavior usually passes easily” (p. 21).

2 See *Time 2* in Table 3 and Fig. 7, in which the data have been normalized to a common scale.

3 A primary advantage of ShinyApps is the use of reactive programming, which circumvents the need for databases or other means of storing data.
