# Correction to: Analyzing the Functional Interdependence of Verbal Behavior with Multiaxial Radar Charts

**DOI:** 10.1007/s40614-024-00418-0

**Published:** 2024-07-23

**Authors:** Lee Mason, Maria Otero, Alonzo Andrews

**Affiliations:** 1 Child Study Center, Cook Children’s Health Care System, 1300 West Lancaster Avenue, Fort Worth, TX 76110 USA; 2 Burnett School of Medicine, Texas Christian University, Fort Worth, TX USA; 3 Department of Behavior Analysis, University of North Texas, Denton, TX USA; 4 Professional and Continuing Education, University of Texas at San Antonio, San Antonio, TX USA

**Correction to: Perspectives on Behavior Science**

10.1007/s40614-024-00404-6

The original article has been updated to include a negative sign before 1 in the formula to read √−1 in the 16th paragraph.

## Data Availability

All data generated or analysed as part of this study are included within the published article.

