# Supplementary material for: Analyzing the Functional Interdependence of Verbal Behavior with Multiaxial Radar Charts
Source: Perspect Behav Sci. 2024 Apr 30;47(2):471–98. doi: 10.1007/s40614-024-00404-6 (PMC11294289; doi:10.1007/s40614-024-00404-6)
Supplement: Supplementary file 1 — Supplementary file1 (DOCX 27 KB) [file 40614_2024_404_MOESM1_ESM.docx]

**Supplementary Material**

Although considered *simple* shape descriptors, the calculations can be somewhat difficult. To facilitate the use of shape descriptors, we have created a Shiny app that allows behavior analysts to enter their own VOX data: [https://behavior.shinyapps.io/VOXanalysis/](https://behavior.shinyapps.io/VOXanalysis). Enter the name of each stimulus, along with a “1” (S^D^) or a “0” (**S**^Δ^) under the corresponding source of control for each verbal response.
